# The data on health locus of control and its relationship with quality of life in HIV-positive patients

**DOI:** 10.1016/j.dib.2018.04.131

**Published:** 2018-05-05

**Authors:** Zahra Mostafavian, Zahra Abbasi Shaye, Arezou Faraj Pour, Golkoo Hosseini

**Affiliations:** a Department of Community Medicine, Mashhad Branch, Islamic Azad University, Mashhad, Iran; b Community Medicine, Treatment Affairs, Mashhad University of Medical Sciences, Mashhad, Iran; c Department of Medical Education, Shahid Beheshti University of Medical Sciences, Tehran, Iran; d Department of Psychiatry, University of Pennsylvania, Philadelphia, PA, USA

**Keywords:** HIV, Health locus of control, Quality of life, Medicine

## Abstract

Locus of control is a concept defined based on social learning theory, and focuses on individuals' beliefs regarding factors that influence their health status. Health Locus of Control (HLC) and its relationship with Quality of Life (QOL) in HIV positive patients in local population were studied. This was a cross-sectional study on 80 HIV-positive patients. Multidimensional Health Locus of Control (MHLC) Scale and Medical Outcome Study Short-Form Health Survey (MOS-SF-36) used to measure patients' HLC and QOL, respectively. Internal, external, and chance HLC mean ± SD scores were 30.31±3.87, 24.17±5.03, and 32.01±4.49, respectively. Positive correlation was found between internal HLC scores and both physical (p <0.001, r = 0.53) and mental quality of life (p <0.001, r = 0.48). Multiple regression analysis showed that internal HLC was the only significant predictor of quality of life. HIV-positive patients who believe their health is mostly influenced by individual's actions and behaviors (internal HLC) showed a higher quality of life. These findings suggest that modifying health locus of control beliefs, hypothetically could influence patients' quality of life.

**Specifications table**

**Table:** 

Subject area	Medicine
More specific subject area	Healthcare
Type of data	Tables
How data was acquired	The data acquired from 80 HIV-positive patients. Multidimensional Health Locus of Control (MHLC) Scale and Medical Outcome Study Short-Form Health Survey (MOS-SF-36) used to measure patients' HLC and QOL, respectively.
Data format	Raw, analyzed
Experimental factors	Convenience sampling method was used. Sample size calculation was done based on a mean formula with Ơ=0.22, z=1.96 and d=0.05.
Experimental features	Normal distribution was assessed by Kolmogorov–Smirnov test. Pearson and Spearman correlation coefficients were used to investigate the correlation between the study variables. To assess the variables affecting quality of life, multiple linear regression method applied for age, gender, education, disease duration, and health locus of control.
Data source location	Mashhad, Razavi Khorasan province, Iran
Data accessibility	Data are included in this article

**Value of the data**

• Health locus of control (HLC) has different types in different people, and is very critical for psychology situation and quality of life of patients, especially those with serious diseases [1], [2], [3].
• There are limited data on the HLC among HIV-positive patients. This article not only provides the above-mentioned data, but also presents the correlation of HLC with quality of life indicators.
• This data shows that HIV-positive patients who believe their health is mostly influenced by individual's actions and behaviors (internal HLC) showed a higher quality of life.
• The data in this study proves that internal health locus of control had a significant correlation with mental and physical of quality of life in the HIV-infected patients.
• The data in this article reveals that paying attention to patients' health locus of control belief and even working on modifying these beliefs could be effective in treatment of HIV-positive patients and their quality of life.

## 1 Data

Study population consisted of 80 HIV positive patients (56 males and 24 females). Mean ± Standard Deviation (SD) of participants' age and infection duration were 38.31 ± 9.15 years and 4.3 ± 3.5 years, respectively. Demographic and clinical data of participants are shown in Table 1. Table 2 demonstrates the descriptive analyses of Quality of Life (QoL) and Health Locus of Control scales among patients. The correlation coefficients of health locus of control dimensions and quality of life domains are shown in Table 3. The internal dimension of HLC showed a statistically significant correlation with both physical and mental dimensions of quality of life. Both correlation coefficients were direct and moderate. Age, sex, and disease duration did not show a statistical significant correlation with physical and mental QoL (p>0.05).

**Table 1:** Demographics characteristics of participants.

**Characteristics**		**Frequency**	**Percentage**
Education	Some elementary school	4	5.0
	Middle school	31	38.8
	High school	30	37.5
	Some college	5	6.2
	College degree or higher	10	12.5
			
HIV infection route	IV drug	41 (39 males, 2 females)	51.2
	Unprotected sex	14 (11 males, 3 females)	17.5
	Marriage	19 (0 males, 19 females)	23.8
	Maternal	1 (1 male, 0 female)	1.2
	Unknown	5 (5 males, 0 females)	6.2

**Table 2:** Dimensions of quality of life and health locus of control among patients.

	**Dimension**	**Mean ± SD**	**Range (Max-Min)**
Quality of life	Physical	59.889±18.32	87.140 - 13.81
	Mental	46.359±17.31	78.570 - 7.14
			
Health locus of control	Internal	30.310±3.87	36 - 15
	Chance	24.170±5.03	35 - 7
	Powerful others	32.010±4.49	36 - 15

**Table 3:** Correlation coefficients of health locus of control and quality of life.

**Quality of life**	**Health locus of control**		
	**Chance**	**Powerful others**	**Internal**
Mental dimension	P=0.146	P=0.143	P=0<001*
	r_p_= −0.164	r_s_= 0.165	r_p_= 0.480
			
Physical dimension	P=0.250	P=0.507	P=0<001*
	r_p_= −0.130	r_s_= 0.075	r_p_= 0.534

r_p_: Pearson correlation coefficient; rs: Spearman correlation coefficient

* P < 0.05 considered statistically significant

Independent sample-t-test did not reveal any significant differences between the two groups physical and mental quality of life scores (p-value = 0.4 and 0.7, respectively). In the ≤ 40 years old group, internal health locus of control showed a statistical significant correlation with higher physical (p = 0.005, r = 0.38) and mental (p = 0.001, r = 0.46) scores of quality of life. Moreover, chance health locus of control showed a statistical significant correlation with lower physical (p=0.03, r=−0.3) and mental (p=0.037, r=−0.29) quality of life. Among patients >40 years old, internal health locus of control showed a statistical significant correlation with higher mental (p<0.001, r=0.67) and physical (p<0.001, r=0.65) quality of life.

Considering gender, independent samples-t-test did not reveal any significant differences between the physical quality of life scores of male (58.57 ± 18.73) and female (62.96 ± 17.3) patients (p-value = 0.33). Likewise, independent samples-t-test did not reveal any significant differences between the mental quality of life scores of male (45.28 ± 18.32) and female (48.85 ± 14.76) patients (p-value = 0.4). For male patients, internal health locus of control showed a statistical significant correlation with higher physical (p<0.001, r=0.55) and mental (p<0.001, r=0.53) quality of life; both correlation coefficients were direct and moderate. In female patients, internal health locus of control showed a statistical significant correlation with only physical quality of life (p=0.014, r=0.32).

Patients were divided based on their HIV infection duration into 2 groups: infection duration less than or equal to 3 years (n = 46) and more than 3 years (n = 34). Independent sample-t-test did not reveal any significant differences between the two groups physical and mental quality of life scores (p-value = 0.8 and 0.7, respectively). In patients with disease duration ≤ 3 years, internal health locus of control showed a statistical significant correlation with mental (p = 0.001, r = 0.48) and physical (p <0.001, r = 0.58) quality of life. Similarly, in patients with disease duration >3 years, internal health locus of control showed a statistical significant correlation with mental (p=0.004, r=0.48) and physical (p=0.004, r=0.47) quality of life. Moreover, powerful others health locus of control showed a statistical significant correlation with physical quality of life (p=0.048, r=0.34).

Multiple linear regression showed that only internal HLC score was significantly predictive (p< 0.001) of 29% of variability in mental and 35% of variability in physical dimension of quality of life (Table 4).

**Table 4:** Linear regression model for predicting the quality of life.

**Quality of life**	**R**	**R Square**	**Adj. R Square**	**p-value**	**β**
Physical quality of life	0.595	0.354	0.291	0<0.001	0.535
Mental quality of life	0.539	0.290	0.221	0<0.001	0.460

β relative contribution of internal HLC score in predicting quality of life

P < 0.05 considered statistically significant

## 2 Experimental design, materials and methods

This cross-sectional study was carried out on 80 patients diagnosed with HIV infection. The patients referred to the Behavioral and Infectious Diseases Consult Center of Mashhad University of Medical Sciences, were recruited by convenience sampling method. Sample size calculation was done based on a mean formula with Ơ=0.22, z=1.96 and d=0.05 [3]. Demographic questionnaire, SF-36 (Short-Form Health Survey), and Multidimensional Health Locus of Control (HLC) scale were used for data collection.

36-item Short-Form Health Survey is a standard self-report measure of quality of life. The 36 questions cover eight health concepts in two main domains of physical and mental health. Physical domain (physical component summary score) consists of four concepts: physical functioning, bodily pain, role limitations due to physical health problems, and general health perceptions. The mental domain (mental component summary score) includes four concepts: energy/fatigue, role limitations due to personal or emotional problems, emotional well-being, and social functioning. Scores for each dimension were summed and converted to zero to 100 according to the scoring board of SF-36, in which zero represents the worst and 100 represents the best condition in the considered scale [2], [4], [5]. SF-36 questionnaire has previously been validated in Iran [6].

MHLC Questionnaire has three forms, including A, B, and C. Form B was used in this study because the researchers were interested in a measure of general health locus of control beliefs and also study population was patients diagnosed with a chronic illness. Moreover, validity and reliability of Persian version of form B have been well studied [7]. Form B of the MHLC consists of 18 items within three subscales of Internal HLC, Powerful Others HLC, and Chance HLC. Each subscale includes six questions with a six-point Likert response. The scale ranges from Strongly Disagree 1 to Strongly Agree 6 [8]. Higher score in one dimension reflects the individual's endorsement of that specific orientation toward health control.

Data analysis was done by the SPSS V.20 software. Normal distribution was assessed by Kolmogorov–Smirnov test. Pearson and Spearman correlation coefficients were used to investigate the correlation between the study variables. Significance was set at 0.05 for all analyses. Authors decided to categorize patients based on their age into two groups: younger or equal to 40 (n=53) and older than 40 years old (n=27). Independent sample-t-test was run to compare the characteristics between these two groups. In order to investigate whether disease duration could influence the quality of life and its association with health locus of control, authors divided the patients based on their HIV infection duration into 2 groups: infection duration less than or equal to 3 years (n = 46) and more than 3 years (n = 34). Independent sample-t-test was used to compare the characteristics between these two groups. In order to assess whether the aforementioned variables could provide a predictive model for quality of life, multiple linear regression method applied for age, gender, education, disease duration, and health locus of control.

## References

[1] Kelly J.A., Murphy D.A., Bahr G.R., Koob J.J., Morgan M.G., Kalichman S.C., Stevenson L.Y., Brasfield T.L., Bernstein B.M., Lawrence J.S. (1993). Factors associated with severity of depression and high-risk sexual behavior among persons diagnosed with human immunodeficiency virus (HIV) infection. J. Health Psychol..

[2] Evans S., Ferrando S.J., Rabkin J.G., Fishman B. (2000). Health locus of control, distress, and utilization of protease inhibitors among HIV-positive men. J. Psychosom. Res..

[3] Preau M., Vincent E., Spire B., Reliquet V., Fournier I., Michelet C., Leport C., Morin M. (2005). Health-related quality of life and health locus of control beliefs among HIV-infected treated patients. J. Psychosom. Res..

[4] Goli F., Scheidt C.E., Gholamrezaei A., Farzanegan M. (2014). The role of locus of control and attributional style in coping strategies and quality of life among Iranian breast cancer and colorectal cancer patients: a pilot study. Int. J. Body Mind Cult..

[5] Luszczynska A., Schwarzer R. (2005). Multidimensional health locus of control: comments on the construct and its measurement. J. Health Psychol..

[6] Montazeri A., Goshtasebi A., Vahdaninia M., Gandek B. (2005). The Short Form Health Survey (SF-36): translation and validation study of the Iranian version. Qual. Life Res..

[7] Moshki M., Ghofranipour F. (2011). Iranian version of Form B of the Multidimensional Health Locus of Control Scales among the youth. J. Clin. Nurs..

[8] Razavi P., Hajifathalian K., Saeidi B., Esmaeeli Djavid G., Rasoulinejad M., Hajiabdolbaghi M., Paydary K., Kheirandish P., Foroughi M., Seyedalinaghi S., Mohraz M., McFarland W. (2012). Quality of Life among persons with HIV/AIDS in Iran: internal reliability and validity of an international instrument and associated factors. AIDS Res. Treat..

